# Metabolite Profiling Reveals the Dynamic Changes in Non-Volatiles and Volatiles during the Enzymatic-Catalyzed Processing of Aijiao Oolong Tea

**DOI:** 10.3390/plants13091249

**Published:** 2024-04-30

**Authors:** Mengcong Zhang, Lixuan Zhang, Chengzhe Zhou, Kai Xu, Guangwu Chen, Linjie Huang, Zhongxiong Lai, Yuqiong Guo

**Affiliations:** 1 Anxi College of Tea Science, College of Horticulture, Fujian Agriculture and Forestry University, Fuzhou 350002, China; zmc.1998@foxmail.com (M.Z.); m15160004025@163.com (L.Z.); chengzhechou@foxmail.com (C.Z.); xukai97@foxmail.com (K.X.); cgw1996@foxmail.com (G.C.); huanglinjie1997@foxmail.com (L.H.); laizx01@163.com (Z.L.); 2 Institute of Horticultural Biotechnology, Fujian Agriculture and Forestry University, Fuzhou 350002, China; 3 Tea Industry Research Institute, Fujian Agriculture and Forestry University, Fuzhou 350002, China

**Keywords:** Aijiao oolong tea, enzyme reaction stage, non-volatile compounds, volatile compounds, metabolomics

## Abstract

The enzymatic reaction stage (ECS) of oolong tea processing plays an important role in the formation of the flavor quality of the oolong tea. To investigate the dynamic changes in the volatile and non-volatile components in the leaves of oolong tea during the ECS, metabolomic studies were carried out using the leaf samples collected at different stages of the ECS of Aijiao oolong tea. Out of the identified 306 non-volatile metabolites and 85 volatile metabolites, 159 non-volatile metabolites and 42 volatile metabolites were screened out as key differential metabolites for dynamic changes during the ECS. A multivariate statistical analysis on the key differential metabolites showed that the accumulations of most metabolites exhibited dynamic changes, while some amino acids, nucleosides, and organic acids accumulated significantly after turning-over treatment. The evolution characteristics of 27 key precursors or transformed VOCs during the ECS of Aijiao oolong tea were clarified, and it was found that the synthesis of aroma substances was mainly concentrated in lipids as precursors and glycosides as precursor pathways. The results revealed the dynamic changes in the flavor metabolites in the ECS during the processing of Aijiao oolong tea, which provided valuable information for the formation of the characteristic flavor of Aijiao oolong tea.

## 1. Introduction

Oolong tea has gained great popularity in the world due to its pleasant floral and fruity aromas [1]. Its quality is predominantly influenced by the tea plant genotype, the cultivation environment, and the processing technology. The fresh leaves of different tea varieties, abundant in different components, undergo a unique processing process that contributes to the unique flavor [2]. The processing steps of oolong tea include plucking, withering, turning-over, fixing, rolling, and drying, which have been classified into an enzymatic reaction (before fixing) and nonenzymatic reaction (after fixing) [3]. During the oolong tea manufacturing process, gradual dehydration and moderate bruising clarify and intensify its special flavor [4]. It has been proposed that the suitability of different varieties to be processed into oolong tea depends on the ratio of terpene volatiles (TVs) to green leaf volatiles (GLVs) during the bruising and wilting of fresh tea leaves, rather than the overall aroma compounds [5]. The initial wounding stress caused by leaf plucking promoted the increase in (E)-β-ocimene content [6]. Ultraviolet-B solar radiation (UV-B) induces the release of volatiles from tea leaves [7] and also induces the production of volatile metabolites from the grape barrier, which protect the tissues from UV-B itself and other abiotic and biotic stresses [8]. Solar withering up-regulates phenylalanine and tryptophan-related synthesis genes and accelerates the chemical conversion of flavonoids [9], and transcriptional changes induced by dehydration stress can induce the conversion of catechins and amino acids [10]. Continuous wounding during the shaking and rocking procedure is the main stress that affects the quality of tea, and the combination with low temperature affects the formation of oolong tea aroma [4]. These findings suggest that the ECS is a key procedure in the manufacture of oolong tea, and will significantly affect the flavor of the produced tea.

The tea leaves of Maoxie, Fujian Shuixian, and Tieguanyin were used as experimental materials to investigate the mechanism of flavor metabolite formation during the ECS [11,12,13]. This study indicates that fresh leaves of different tea varieties have different flavor metabolite transformation mechanisms during processing due to differences in their morphological structure and contents composition. Fujian, the origin-place and main production area of oolong tea, is mainly divided into Southern Fujian’s and Northern Fujian’s oolong tea production areas. Jianou City belongs to the oolong tea production area of Northern Fujian, which is the hometown of the historically famous tea “Dragon and Phoenix Group Tea” and “Beiyuan Tribute Tea”, and also one of the largest oolong tea producing areas in the country. As the main plant cultivar in Jianou, the tea made from it has a strong peach aroma and mellow taste [14]. The processing technology of Aijiao oolong tea is characterized by strong sunlight withering, light shaking, and the heavy discoloration of turning-over, which contributes to the unique and attractive flavor of Aijiao oolong tea [15]. However, the specific effects of withering and turning-over on the flavor quality of Aijiao oolong tea have not been revealed.

The aim of this work was to explore the dynamics changes during the ECS and elucidate the contribution of flavor quality to Aijiao oolong tea by the ECS. Non-targeted metabolomics is a new analytical tool with high throughput and sensitivity, which can simultaneously determine the composition of multiple substances and comprehensively analyze the changes in metabolites [16]. It is widely used in the field of tea. Therefore, this study uses the Aijiao oolong fresh tea leaves as the raw material to process oolong tea and set four processes for observation (fresh leaves, withering, turning-over, and drying). Metabolites and aroma substances of these tea samples were detected using LC-MS, GC-MS, and other technologies. Multiple statistical analysis methods were used to elucidate the dynamic evolution of volatile compounds and non-volatile compounds, and the key metabolic pathways of volatiles biosynthesis during the enzyme reaction stage of oolong tea. This result will provide new insight into the dynamic changes in metabolites in the ECS during the processing of Aijiao oolong tea, as well as contribute to optimizing the processing to enhance the flavor quality of oolong tea.

## 2. Results and Discussion

### 2.1. Evolution of Flavor Characteristics during the ECS of Aijiao Oolong Tea

**Figure 1:**
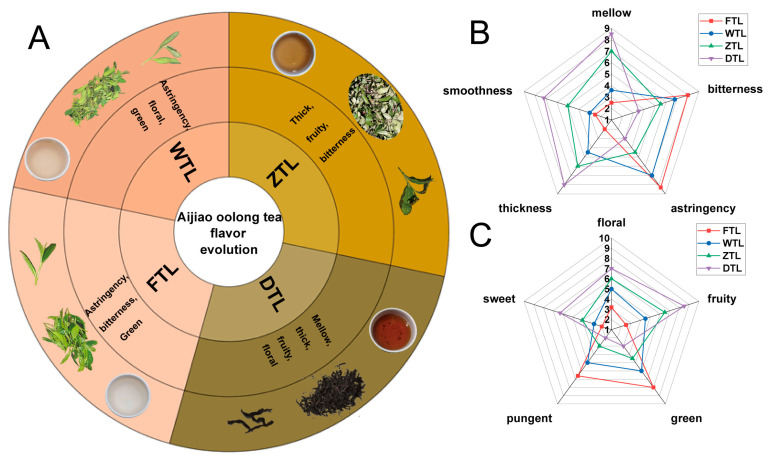
The evolution of flavor characteristics during the ECS of Aijiao oolong tea. (**A**) Flavor wheel for the ECS of Aijiao oolong tea. The three images in the same column are the processed fresh leaves with one bud and three leaves, a freeze-dried sample, and a tea infusion of a freeze-dried sample. (**B**) The radar map of changes in taste attributes. (**C**) The radar map of changes in aroma attributes.

Figure 1A shows the phenotypic changes in fresh leaves and tea infusions, and the evolution of their taste and aroma senses, during the ECS of Aijiao oolong tea. The evolution of taste and aroma was reflected in the changes in the intensity of each sub-attribute in Figure 1B,C. The bitterness, astringency green, and pungent dominated the flavor characteristics of the tea infusion at the FTL and WTL. At ZTL, mellow, floral, and fruity became dominant. After drying, the flavor of Aijiao oolong tea was dominated by sweetness, umami, mellow, and smoothness, but there were still moderate levels of astringency and bitterness. Overall, during the ECS of Aijiao oolong tea, the bitterness, astringency green, and pungent were significantly reduced, while attributes such as mellow, thickness, floral, and fruity were significantly increased, ultimately leading to a taste dominated by fruity and mellow. Zhou et al. [17] found that the characteristic taste of white tea was formed in the withering process, and the fresh leaves had a strong bitter-astringent flavor. The unique turning-over process takes up most of oolong tea’s processing time and creates massive charming volatiles [18]. These may explain the changes in flavor during the ECS of Aijiao oolong tea.

### 2.2. Analysis of Non-Volatile Metabolites during the ECS of Aijiao Oolong Tea

The metabolite identification was conducted by comparing their mass-to-charge values, retention times, and fragmentation patterns with the authentic standards or by searching against the public databases (OTCML, MoNA, BioDeepDB, MetaDNA, mzCloud, OTCML, and GNPS). A total of 306 non-volatile metabolites were detected (Table S1), including 48 amino acids and their derivatives, 45 flavonoids, 40 lipids, 34 sugars and their glycosides, 32 organic acids, 32 nucleotides and their derivatives, 24 phenolic acids, 17 alkaloids, 12 tannins, 9 lignans and coumarins, and 13 other metabolites. The overlay analysis of the base peak chromatograms (BPCs) in positive and negative ion modes showed that the data obtained in this study have the characteristics of strong mass detection signal, large peak capacity, and high resolution [19] (Figure 1A,B). 

**Figure 2:**
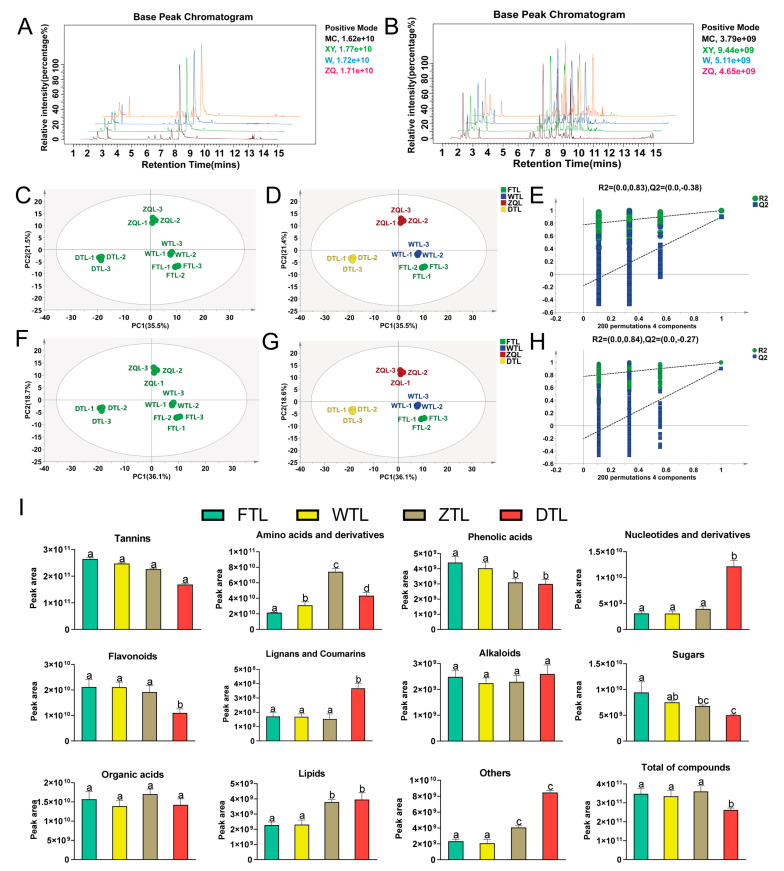
(**A**) Total ion current of four group samples, as revealed by mass spectrometry detection in positive ionization mode. (**B**) The total ion current of four group samples, as revealed by mass spectrometry detection in negative ionization mode. (**C**) The PCA score scatter plot of all non-volatile metabolites in positive ion mode. (**D**) The PLS-DA score scatter plot of all non-volatile metabolites in positive ion mode (R2X = 0.722, R2Y = 0.996, and Q2 = 0.88). (**E**) The permutation test for PLS-DA model validation in positive ion mode. (**F**) The PCA score scatter plot of all non-volatile metabolites in negative ion mode. (**G**) The PLS-DA score scatter plot of all non-volatile metabolites in negative ion mode (R2X = 0.714, R2Y = 0.993, and Q2 = 0.895). (**H**) The permutation test for PLS-DA model validation in negative ion mode. (**I**) The dynamic changes in non-volatile compound types during the ECS of Aijiao oolong tea. The various superscripts show significant differences (*p* < 0.05).

To further explore the changing trends of non-volatile metabolites during processing, multivariate statistical analysis which is based on all non-volatile metabolites was conducted. The PCA score plot showed a clear separation in the samples of FTL, WTL, ZTL, and DTL, in positive and negative ion modes (Figure 2C,F), which indicated a significant difference in non-volatile metabolites among the four sets of samples. In contrast to PCA, PLS-DA is a supervised analysis method that can perform better both classification and feature selection to investigate the differences in the samples (Figure 2D,G). As shown in Figure 2D (t[1] = 35.5%, t[2] = 21.4%) and G (t[1] = 36.1%, t[2] = 18.6%), the fresh tea leaves are on the upper left side of the figure, the withered tea leaves are on the left center, the turning-over tea leaves are on the lower left, and the dried tea leaves are on the right center, thereby indicating a clear separation between the four groups samples, and in particular between the withered and turning-over, as well as between the turning-over and dried leaves. In contrast, there is a small difference between the fresh and withered leaves. Also, the permutation plot of PLS-DA showed that the model had an effective predictive ability and without overfitting (Figure 2E,H). As shown in Figure 2I, the total amounts of non-volatile metabolites exhibited the following trend: ZTL > FTL > WTL > DTL, and 11 types of the metabolites exhibited significant changes during processing (*p* < 0.05). Among them, the quantities of amino acids and their derivatives and organic acids initially increased prior to reaching a peak during turning-over, and then subsequently decreased. The tannins, flavonoids, phenolics acids, and sugars showed a downward trend, while alkaloids, nucleotides and derivatives, lignans and coumarins, lipids, and others showed an upward trend. Overall, the amino acids and their derivatives, alkaloids, nucleotides and their derivatives, lignans and coumarins, organic acids, and lipids underwent the most significant changes after turning-over, fixation, and drying, which is consistent with the PCA results. The dynamic changes in these metabolites were mainly caused by the enzyme-catalyzed reactions induced by multiple stresses in the enzyme reaction stage, which were subsequently consolidated by the thermal effects occurring during the fixing and drying steps of oolong tea manufacture, which are congruous with the previous report [20].

### 2.3. Differential Non-Volatile Metabolites Screening

To clarify the effect of the major process on the non-volatile metabolites at the various stages of Aijiao oolong tea processing, the key distinctions between the processes were investigated. More specifically, the orthogonal partial least squares discrimination analysis (OPLS-DA) of FTL vs. WTL, WTL vs. ZTL, and ZTL vs. DTL were performed (Figure S1), according to the principle of VIP > 1 and fold change ≥2 or ≤0.5 for the screening of the different metabolites. A total of 159 different substances were screened (Table S2); the number of different substances were increasing with the process of the enzyme reaction stage, with the most abundant different substances observed in ZTL vs. DTL (59 up-regulated and 57 down-regulated). Only 22 different substances (18 up-regulated and 4 down-regulated) were observed at the early stage of the enzyme reaction stage (FTL vs. WTL), suggesting the enzymatic-catalyzed conversion of the non-volatile metabolites is a cumulative process, and then the thermal effect in firing and drying process promoted the alterations of the non-volatiles.

**Figure 3:**
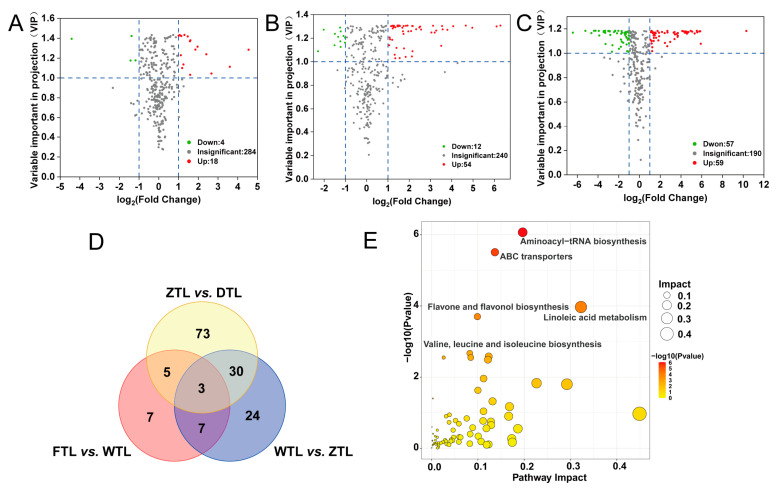
Differential non-volatile metabolites present in Aijiao oolong tea of FTL vs. WTL, WTL vs. ZTL, and ZTL vs. DTL. (**A**–**C**) The volcano plot of the differential non-volatile metabolites of FTL vs. WTL, WTL vs. ZTL, and ZTL vs. DTL. (**D**) The Venn diagram of the differential non-volatile metabolites of FTL vs. WTL, WTL vs. ZTL, and ZTL vs. DTL. (**E**) The pathway clustering analysis on the differential non-volatile metabolites of FTL vs. WTL, WTL vs. ZTL, and ZTL vs. DTL.

As shown in the Venn diagram (Figure 3D), both common and unique metabolites exist between the different comparison groups. More specifically, FTL vs. WTL, WTL vs. ZTL, and ZTL vs. DTL possess three common substances (i.e., L-Tyrosine, Cyclic AMP, and Apigenin), which are simultaneously impacted by the water loss stress of withering, the mechanical injury of turning-over, and the heat of fixation and drying. All the differential non-volatile metabolites were subjected to the KEGG pathway enrichment analysis by using the online platform BioDeep (https://www.biodeep.cn/, accessed on 1 December 2023). As shown in Figure 3E, Aminoacyl-tRNA biosynthesis, ABC transporters, linoleic acid metabolism, Flavone and flavonol biosynthesis, Tropane, piperidine and pyridine alkaloid biosynthesis, phenylalanine, tyrosine and tryptophan biosynthesis, purine metabolism, and the biosynthesis of unsaturated fatty acid metabolism were significantly enriched, and were highly correlated with the dynamic changes in key metabolites. The differential non-volatile metabolites significantly enriched in the phenypropanoid biosynthesis, flavonoid biosynthesis as well as the alpha-linolenic acid metabolism demonstrated (Figure 3E), which were probably a spontaneous response to alleviate the intracellular injury induced by environmental stresses [21]. The biosynthesis of aminoacyl-tRNAs and amino acid metabolism are also highly focused in pathway analysis, probably because amino acid metabolism minimizes adverse conditions by involving the synthesis of the secondary metabolites and signaling molecules [22].

### 2.4. Dynamic Evolution of the Differential Non-Volatile Metabolites during the ECS of Aijiao Oolong Tea

#### 2.4.1. Amino Acids and Their Derivatives

**Figure 4:**
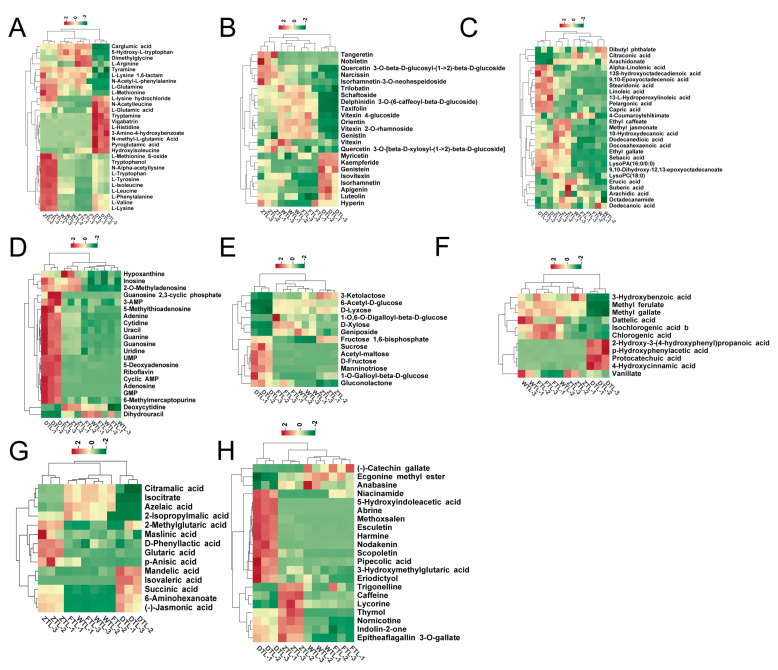
Heat map of the levels of the 159 differential non-volatile metabolites during different tea processes. Note: amino acids and their derivatives (**A**), flavonoids (**B**), lipids (**C**), nucleotides and derivatives (**D**), sugars (**E**), phenolic acids (**F**), organic acids (**G**), tannins, lignans and coumarins, alkaloids, and others (**H**), respectively.

Amino acids and their derivatives have taste characteristics such as umami, sweetness, kokumi, and bitterness, which contribute strongly to the taste of tea [23]. The dynamic evolution of the content of 29 amino acids and their derivative differential metabolites during main processing is shown in Figure 4A. After withering, L-leucine, L-phenylalanine, L-tyrosine, and N-acetyl-L-leucine contents significantly increased by 2.5-, 2.1-, 2.1-, and 2.2-fold, respectively, whereas L-arginine content significantly decreased, which is consistent with the results of others [24]. L-phe is an important product in the shikimic acid metabolism pathway, which is used as a precursor to generate aroma substances such as benzaldehyde, benzyl alcohol, and benzoic acid through enzymatic and non-enzymatic reactions [25]. After the turning-over process, the levels of 11 metabolites, including L-aspartic acid, L-methionine, L-lysine, L-tyrosine, and so on, were significantly up-regulated. Among those L-aspartic acid was one of the main contributing components to the fresh taste of tea infusion, as well as the levels of tyrosine and phenylalanine were significantly increased during both withering and turning-over, which were found to be potential markers for oolong tea processing in the earlier study [24]. According to previous studies, proteolysis may be the reason for an increase in many minor amino acids following turning-over [24,26,27]. From the end of the turning-over to the drying process, 8 metabolites were significantly up-regulated in abundance and 15 metabolites were significantly down-regulated in abundance, and most of them showed a significant decrease after drying, as the high temperature inactivates the enzyme and converts amino acids to aromatic compounds through the Maillard reaction [28]. Perhaps this explains why the concentrations of the amino acids dropped sharply during this process.

#### 2.4.2. Flavonoids and Lipids

As mentioned earlier, flavonoids, which are important components of tea infusion taste and color, have lower taste thresholds than catechins, which has increased the attention of researchers recently. In this study, the dynamic change trends of the content of 23 flavonoid differential metabolites showed noteworthy differences during tea processing. As shown in Figure 4B, the content of nearly half of the substances was increasing, the other half was decreasing, and a small number of substances fluctuated up and down. Among these substances, the contents of Apigenin, Isorhamnetin, Isovitexin, Luteolin, and Vitexin increased significantly by 3-, 12.1-, 2.4-, 3.6-, and 2.3-fold, respectively, following withering. It is possible that during withering, multiple stresses such as sunlight radiation, high temperature, or water dissipation induce a large accumulation of reactive oxygen species (ROS), and these flavonoids with antioxidant properties are synthesized to eliminate the ROS generated by unfavorable environments. After turning-over, the level of 5 metabolites was significantly up-regulated and 2 substances was significantly down-regulated, while the abundance of 4 metabolites was significantly up-regulated and 12 metabolites was significantly down-regulated after drying. The content of the vast majority of flavonoid glycosides decreased significantly, which may be due to the heat instability of glycosidic bonds, which degrade into flavonoids and sugar monomers after high-temperature action. Flavonoid glycosides have an extremely low taste threshold, which is the main source of the pronounced astringency and bitterness of tea infusion. The greatly reduced content of flavonoid glycosides may be an important reason for the enhanced taste quality of oolong tea [29,30].

Lysophospholipids, as major components of biological membranes, are signaling molecules associated with various unfavorable environmental responses [31]. In this study, we found that the contents of fatty acid derivatives such as lysophosphatidylcholine (LysoPC(18:0)) and lysophosphatidylethanolamine (LysoPA(16:0/0:0)) were significantly up-regulated after the turning-over stage, as fatty acid derivatives may be involved in the regulation of leaf stress signaling and contribute to the formation of the oolong tea flavor [11]. Methyl jasmonate was derived from fatty acid metabolism and were also increased during the withering and turning steps. This result is consistent with previous studies that jasmonic acid (JA) derivatives are produced in abundance as initiators, triggering downstream biological processes in response to water loss and mechanical wounding during the enzyme reaction stage [32]. The number of the specific significant lipid metabolites was two, eight, and nine in FTL vs. WTL, WTL vs. ZTL, and ZTL vs. DTL, respectively. With the exception of Ethyl caffeate, other free fatty acids (10-Hydroxydecanoic acid, 9,10-Epoxyoctadecenoic acid, Citraconic acid, Ethyl caffeate, linoleic acid, and Stearidonic acid) are the common significantly metabolites between WTL vs. ZTL and ZTL vs. DTL. The mainly free fatty acids, lysophosphatidylcholine, and lysophosphatidylethanolamine were significantly up-regulated after turning-over, as fatty acid derivatives are potentially involved in the regulation of stress signaling in the leaves and contribute to the development of flavor over the enzyme reaction stage of oolong tea production [11]. Nine kinds of free fatty acids were significantly up-regulated after fixation and drying, which can be related to high temperatures and can considerably promote lipid conversion to free fatty acids [30].

#### 2.4.3. Nucleotides and Derivatives and Sugars

Nucleotides and derivatives are important secondary metabolites in tea plants, which have an umami flavor and can improve the taste of tea infusion. 5-Deoxyadenosine, 5-Methylthioadenosine, Adenine, and Adenosine were significantly up-regulated after the turning-over treatment, while GMP and Guanosine 2,3-cyclic phosphate were significantly down-regulated after the turning-over treatment, and then significantly accumulated after drying. Earlier studies have shown that fixing is the key to enhancing the levels of most nucleotides and their derivatives [33]; the degradation of RNA is believed to be responsible for the increased levels of nucleosides and nucleotides [34]. Earlier research has shown that sugar compounds provide little contribution to the sweetness intensity of the tea but can serve to optimize the taste of the tea broth [35]. Notably, the metabolites such as 6-Acetyl-D-glucose, D-Fructose, Geniposide, and Gluconolactone displayed a decreasing trend during the enzyme reaction stage of Aijiao oolong tea. These carbohydrate derivatives potentially function as precursors or signaling molecules over metabolic processes in response to adverse environmental conditions, such as water deficit, osmotic stress, or extreme temperature in plants [36]

#### 2.4.4. Phenolic Acids and Organic Acids

Phenolic acids may contribute to the bitterness and astringency of tea [37]. Chlorogenic acid, Dattelic acid, and Isochlorogenic acid did not change significantly during the enzymatic reaction, but decreased to 47%, 25%, and 36%, respectively after drying, which promotes the formation of the sweet and mellow taste of Aijiao oolong. Organic acid is a water-soluble compound in tea, which is one of the important components of tea aroma and taste, especially for the formation of sour taste [38]. After turning-over, (-)-Jasmonic acid, 6-Aminohexanoate and Glutaric acid contents significantly increased by 11.7-, 25.3-, and 8.7-fold, respectively. Most of the organic acids (2-Isopropylmalic acid, Azelaic acid, and Isocitrate) were significantly down-regulated after drying; this is consistent with the conclusion that the number of organic acids decreases as leaf freshness decreases [38]. Lv et al. analyzed the chemical components of the taste quality of Pu ‘er tea and found that the content level of organic acids was markedly negatively correlated with the score of taste quality [39].

#### 2.4.5. Tannins, Lignans and Coumarins, Alkaloids and Others

Tannins are important polyphenols in tea which may contribute to the astringent taste of tea [40]. After withering, Epitheaflagallin 3-O-gallate increase was 3.3 fold, but the (-)-Catechin gallate is reduced to 20%, which indicated that the tannins were greatly affected by the water loss stress during the withering process. Alkaloids are a class of nitrogenous organic compounds, and purine alkaloids are the main alkaloids in tea, including caffeine, theobromine, and theophylline, which play an important role in the bitterness and astringency of tea infusion [35]. After the turning-over treatment, the content of most alkaloids was significantly increased, especially the content of caffeine increased by 4.4 fold. Caffeine is an abundant metabolite in tea and is considered to be associated with bitterness [41]. In addition, some lignans, coumarins, and others showed significant changes during the enzymatic reactions in the processing of Aijiao oolong tea (Figure 4H).

### 2.5. Analysis of VOCs during the ECS of Aijiao Oolong Tea

**Figure 5:**
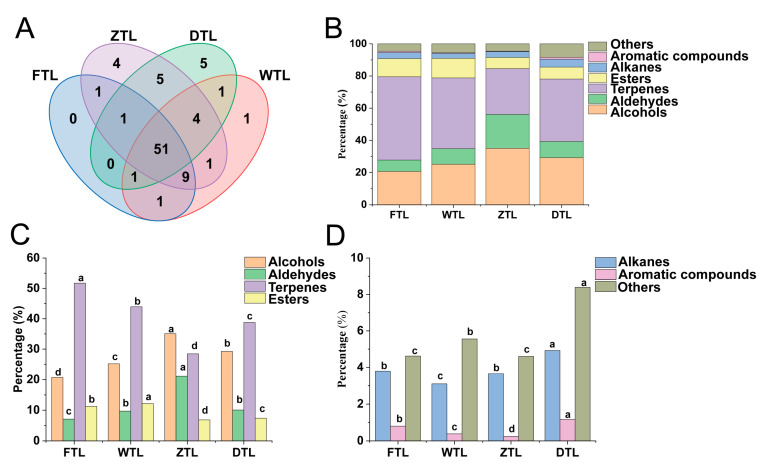
The change in the VOC content during Aijiao oolong tea processing: (**A**) The Venn diagram of the differential volatile metabolites between four samples. (**B**) The trend chart of the proportion of the different types of VOCs during Aijiao oolong tea processing. (**C**,**D**) The variation in the proportion of the different types of VOCs during Aijiao oolong tea processing. The various superscripts show significant differences (*p* < 0.05).

A total of 85 VOCs were determined from the leaf samples collected from the ECS stage of Aijiao oolong tea production, mainly including 23 alcohols, 11 aldehydes, 13 esters, 15 terpenes, 10 alkenes, 3 aromatic compounds, and 10 others (Table S3). Alcohols, aldehydes, terpenes, and esters are the main types of aroma substances in the main processing of oolong tea. Among them, terpenes are one of the main components in FTL (Figure 5C,D), which is more than 50% and reaches the highest proportion (51.37%). After the turning-over step, the proportion of alcohols and aldehydes increased significantly (Figure 5C), which is consistent with the results of existing research [6]. Meanwhile, terpenes, esters, and aromatic compounds decreased drastically (*p* < 0.05). During the turning-over step, green leaves lose water, cell membrane permeability is enhanced and the structure and function of organelles change, amino acids, alcohols, and aldehydes are produced through starch, protein hydrolysis, and unsaturated fatty acid oxidation, thus forming the unique aroma of oolong tea [42]. After the drying process, alkanes, aromatic, and other categories of compounds increased significantly. Therefore, different processing processes have significant and different influences on the VOCs and the proportion of aroma components in the final tea samples, among which turning-over and drying are the key processes affecting the aroma transformation of tea [43].

**Figure 6:**
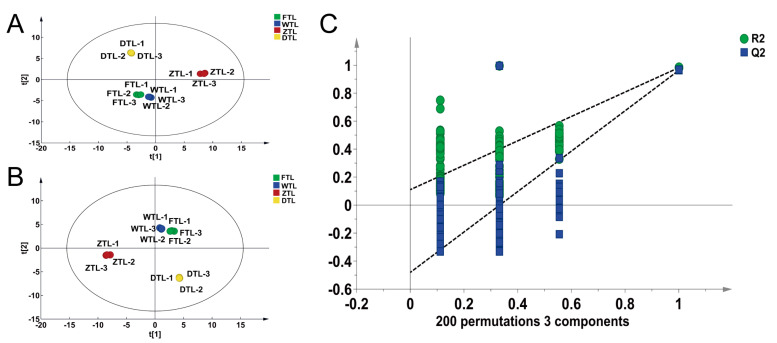
(**A**) PCA score scatter plot composed of all the VOCs. (**B**) The dynamic trajectory plot of the VOCs, drawn using the scattering of the PLS-DA scores. (**C**) Permutation test plots of all the VOCs.

To further explore the different trends of VOCs during processing, a multivariate statistical analysis which is based on 51 common VOCs was conducted. As can be seen from the PCA score plot, there is a clear separation in the samples of FTL, WTL, ZTL, and DTL (Figure 6A). Subsequently, the PLS-DA model was used to confirm whether the VOCs were affected by the processing (Figure 6B). As shown in Figure 6B (t[1] = 51.9%, t[2] = 38.5%), the FTL and WTL are on the upper left side of the figure, the ZTL sample is on the lower left, and the DTL is on the right bottom, thereby indicating a clear separation between the four groups samples, and in particular between the withered and turning-over, as well as between the turning-over and dried leaves. In contrast, there is a small difference between the fresh and withered leaves. Cross-validation analysis showed that the OPLSDA models were reliable (Figure 6C).

### 2.6. Differential VOC Screening

**Figure 7:**
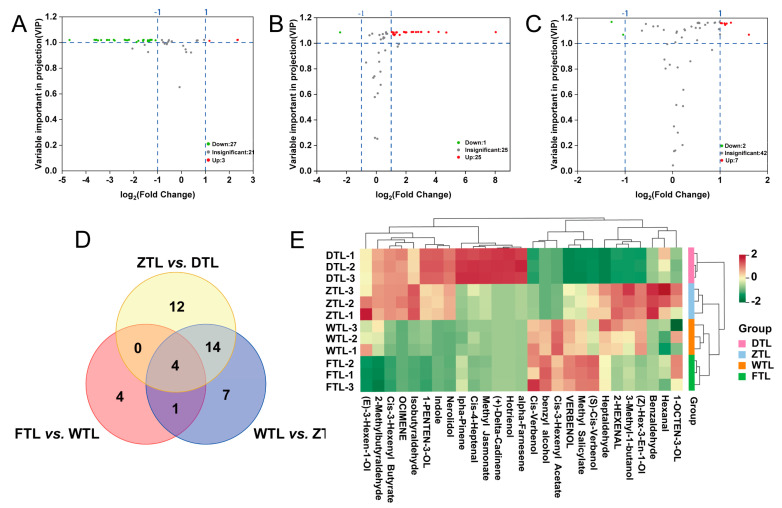
Differential VOCs present in Aijiao oolong tea of FTL vs. WTL, WTL vs. ZTL, and ZTL vs. DTL. (**A**–**C**) The volcano plot of the differential VOCs of FTL vs. WTL, WTL vs. ZTL, and ZTL vs. DTL. (**D**) The Venn diagram of the differential VOCs of FTL vs. WTL, WTL vs. ZTL, and ZTL vs. DTL. (**E**) The heat map of the key differential VOCs and their related substances during the processing of Aijiao oolong tea.

To determine whether the VOCs were largely affected by the processing of Aijiao oolong tea, partial least squares discrimination analysis (PLS-DA) of FTL vs. WTL, WTL vs. ZTL, and ZTL vs. DTL was performed (Figure S2) according to the principle of VIP > 1 and fold change ≥2 or ≤0.5 for the screening of the different VOCs. A total of 42 different substances were screened (Table S4), out of which 30 differences were recorded between ZTL and DTL (Figure 7A, 3 up-regulated and 27 down-regulated), 26 differences were observed between ZTL and WTL (Figure 7B, 25 up-regulated and 1 down-regulated), and 9 differences were observed between WTL and FTL (Figure 7C, 7 up-regulated and 2 down-regulated).

As shown in the Venn diagram (Figure 7D), both common and unique metabolites exist between the different comparison groups. More specifically, FTL vs. WTL, WTL vs. ZTL, and ZTL vs. DTL possess four common substances (i.e., 2-Methylbutyraldehyde, (Z)-Hex-3-En-1-Ol, Cis-3-Hexenyl Butyrate, and α-Famesene), which are simultaneously impacted by the water loss stress of withering, the mechanical injury of turning-over, and the heat of fixation and drying. The 12 VOCs that were unique to ZTL vs. DTL were only affected by the fixation and drying temperature, while 7 VOCs were only affected by mechanical damage during turning-over process; 4 metabolites were only affected after the withering process.

### 2.7. Evolution of Key Differential VOCs and Their Related VOCs during the ECS of Aijiao Oolong Tea

Combined with metabolism laws and the typical transformation pathway of VOCs in tea [44,45], the evolution characteristics of the 27 key precursors or transformed VOCs during Aijiao oolong tea processing were clarified based on the 42 differential VOCs (Figure 7E). According to the categories of the volatile metabolite precursors, there were 14 fatty acid-derived VOCs, 7 glycoside-derived VOCs, 2 carotenoid-derived VOCs, and 4 amino acid-derived VOCs, which indicates that lipids and glycosides are key precursor categories for aroma formation in the processing of Aijiao oolong tea (Supplementary Table S5).

#### 2.7.1. Fatty Acid-Derived VOCs

**Figure 8:**
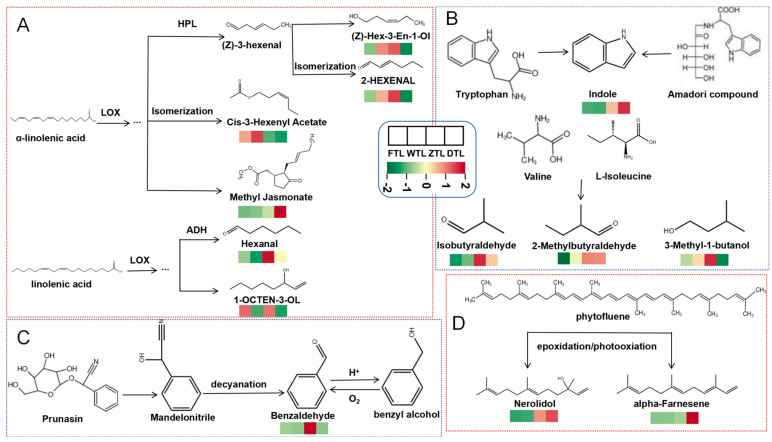
Evolution characteristics of key differential VOCs and related substances obtained from different sources during Aijiao oolong tea processing: (**A**) fatty acid-derived VOCs, (**B**) amino acid-derived VOCs, (**C**) glycoside-derived VOCs, and (**D**) carotenoid-derived VOCs.

Lipids are important precursors of volatile compounds produced in green tea and black tea processing [30,46]. Due to the degradation of unsaturated or saturated fatty acids, they can form the corresponding volatile compounds containing C6-9. Figure 8A shows the pre- and post-transformation relationship of lipid metabolites during the processing of Aijiao oolong tea. Under the action of lipoxygenase and hydroperoxide lyase, after turning-over the (Z)-Hex-3-En-1-Ol, 2-HEXENAL, and Hexanal formed from α-linolenic acid and linoleic acid showed a sharp upward trend, whereas the contents of (Z)-Hex-3-En-1-Ol, 2-HEXENAL, and 1-OCTEN-3-OL decreased after drying. Informed research have shown that hexanal is derived from linoleic acid [44]. Thus, in the drying stage, under the influence of isomerization and REDOX reactions, the oxidation of fatty acids can produce a certain amount of aldehydes (such as hexanal) and low-boiling-point esters (such as methyl jasmonate) under the combined action of heat and enzymes [47]. In addition, high-boiling-point compounds, such as hexanal and 1-penten-3-ol, increased significantly during the drying process (*p* < 0.05), likely because the leaf tissue is broken due to mechanical damage and dehydration in the process of turning-over, resulting in a large amount of unsaturated fatty acids produced [48].

#### 2.7.2. Amino Acid-Derived VOCs

Amino acid-derived volatiles are usually derived from the Strecker reaction of amino acids and the Maillard reaction (Tea aroma formation) between amino acids and sugars. Amino acid-derived volatiles were identified in this study (Figure 8B). Among them, Isobutyraldehyde, 2-Methylbutyraldehyde, and 3-Methyl-1-butanol increased significantly after the stage of turning-over (*p* < 0.05); as these three volatiles are usually floral, fruity, or sweet, the accumulation of these may contribute to the formation of floral, fruity or sweet oolong tea. After drying, indole content increased significantly. Indole is the key aromatic active compound in oolong tea [49]. It can be obtained by the Amadori product of L-tryptophan under pyrolysis conditions and oxidized by tryptophan indole lyase [44]. In addition, the indole content increased slightly after the greening process, which suggested that the continuous damage stress induced the formation and accumulation of the indole [50].

#### 2.7.3. Glycoside-Derived VOCs

Glycosides play an important role in the aroma of oolong tea, which is closely related to the formation of volatile terpenoids. There are a large number of volatile components in the form of glycosides in the fresh leaves. In the process of tea processing, they are easy to be hydrolyzed by endogenous glycosidase to produce aromatic alcohols (such as benzyl alcohol) and fatty alcohols with fruity and sweet flavors (Figure 8C). Benzaldehyde and OCIMENE increased significantly during the turning-over process, which may be related to the increased cell breakage rate under external force, high water content, and the consumption of precursor substances [51]. The content of benzaldehyde, a key intermediate of VBs, did not change significantly after withering, but increased significantly after the turning-over treatment, indicating that the accumulation of benzaldehyde had no significant effect under light or heat stress during the withering process but significantly responded to mechanical stress during turnover. The dynamic change in the benzyl alcohol content in the withering stage was exactly opposite to the dynamic change in the benzaldehyde content, which was in line with the conversion rule of trans-cinnamic acid (PAL branch), which indirectly indicated that the withering and greening treatment were effective measures to gradually promote the formation of VBs in Aijiao oolong tea [25]. After drying, the contents of the volatile components such as Methyl Salicylate and phenyl ethanol decreased significantly, and the new volatile components such as Hotrienol and (+)-Delta-Cadinene were produced correspondingly. Many glycoside volatiles are lost during drying which is consistent with previous findings [52]. It is speculated that in high-temperature environments, alcohol, aldehydes, and glycoside volatile components with low boiling points and high volatility are isomerized, oxidized, and undergo other reactions to produce high-boiling-point volatile compounds with baked, fruity, and fresh aromas [51].

#### 2.7.4. Carotenoid-Derived VOCs

Under the action of photooxidation, carotenoids produce nerolol, α-Farnesene, and other floral and fruity aroma substances, which are important precursors for the formation of tea aromas such as green tea and oolong tea [44]. These substances are all formed by the photooxidation (solar wilt and solar drying) reactions of plant fluorescein (Figure 8D). The content of Nerolidol and alpha-Farnesene increased sharply in the process of turning-over, and basically retained and slightly increased in the drying process. Previous studies have shown that Nerolidol tertiary alcohol content increases under various stresses [52]. Thus, the significant accumulation of carotenoid-derived volatiles may contribute to the formation of sweetness and floral fragrances in oolong tea to a large extent.

## 3. Materials and Methods

### 3.1. Chemicals

The methanol and acetonitrile (LC-MS grade) were purchased from Thermo Fisher Scientific Co. (Fair Lawn, NJ, USA). 2-Chlorophenylalanine (98.5%), ammonium formate (≥99.0%), and formic acid (LC-MS grade) were obtained from Aladdin Biochemical Technology Co., Ltd. (Shanghai, China), Sigma-Aldrich (St. Louis, MO, USA), and TCI Chemical Industry Development Co., Ltd. (Shanghai, China), respectively. Ultrapure water used in this study was obtained from a Milli-Q purification system (Millipore, Billerica, MA, USA).

### 3.2. Materials

The fresh tea leaves (*Camellia sinensis* var. Aijiao oolong) were plucked for the purpose of this study, following the picking standards of collecting a bud with three or four leaves [29]. The tea leaves were collected in April 2023 in Jianou City (118°32 E, 27°03 N). Subsequently, the fresh leaves were processed into Aijiao oolong tea by the professional tea masters of Jianou Chenglong Tea Factory according to the traditional manufacturing method [20]. Samples were collected after each process step during the whole manufacturing process. Specifically, a total of 4 types of samples in triplicate were taken from the processing steps, namely, fresh tea leaves (FTL), withered tea leaves (WTL), turning-over tea leaves (ZTL), and dried tea leaves (DTL). The samples collected at every stage were frozen immediately with liquid nitrogen and stored at −80 °C for further study.

### 3.3. Sensory Evaluation

Evaluation and scoring were performed by eight professional and trained sensory recognition panelists (four females and four males, 30 to 50 years old) from the Fujian Agriculture and Forestry University, according to the method used in the authors’ previous study [53], with some modifications. Briefly, tea infusions were prepared according to national standards (GB/T 23776-2018 [54]): each tea sample (5 g) was brewed with 110 mL boiling water for 5 min and discarded, after which the tea infusions labeled with a three-digit code were presented to each panelist in a randomized order. Then, the intensity values (0–10), taste descriptors (smoothness, mellow, thickness, astringency, and bitterness), and aroma descriptors (floral, fruity, green, sweet, and pungent) of each sample infusion were subjected to a sensory test by the six panelists. A scale from 0 to 10 (where 0 was none or no perception and 10 was extremely strong) as described in a previous study was used to symbolize the intensity values.

### 3.4. LC-MS Analysis of Non-Volatile Metabolites

The non-volatile compound extraction for the LC-MS analysis was carried out according to our previously published protocol [19]. Three replicates were prepared for each sample. The 0.6 mL 2-chlorophenyl alanine (4 ppm) methanol (−20 °C) was added into a 2 mL EP tube containing 200 mg (±1%) of tea samples and smashed using a tissue grinder. After centrifugation (10 min, 12,000 rpm, and 4 °C), the supernatant was collected, and filtered through a 0.22 µm membrane for LC-MS detection.

Chromatographic separation was used with an ACQUITY UPLC^®^ HSS T3 (150 × 2.1 mm, 1.8 µm, Thermo Fisher Scientific, Waltham, MA, USA) column maintained at 40 °C. The temperature of the autosampler was 8 °C. The mobile phases were positive 0.1% formic acid in water (C)-0.1% formic acid in acetonitrile (D) and negative ions 5 mM ammonium formate in water (A)-acetonitrile (B) at a flow rate of 0.25 mL/min. The injection of 2 μL of each sample was performed after equilibration. The gradient elution procedure in positive ion mode was 0~1 min, 2% D; 1~9 min, 2~50% D; 9~12 min, 50~98% D; 12~13.5 min, 98% D; 13.5~14 min, 98~2% D; and 14~20 min, 2% D. The gradient elution procedure in negative ion mode was 0~1 min, 2% B; 1~9 min 2~50% B; 9~12 min, 50~98% B; 12~13.5 min, 98% B; 13.5~14 min, 98~2% B; and 14~17 min, 2% B. The chromatographic condition was used according to the one established previously [18]. Mass spectrometry was executed by a Thermo Q Exactive Focus mass spectrometer (Thermo Fisher Scientific, USA) equipped with a heated electrospray ionization (ESI) probe, and the parameters were set as follows: Electrospray ionization was performed in both positive and negative ionization modes with a spray voltage of 3.5 kV and −2.5 kV, respectively. The flow rate of sheath gas and auxiliary gas was 30 and 10 arbitrary units, respectively. The capillary temperature was 325 °C. The resolution of full scan MS was set as 60,000 and the mass scan range was mass–charge ratio (*m*/*z*) 100–1000. Data-dependent acquisition (DDA) MS/MS experiments were performed with a high-energy collision–dissociation (HCD) scan. The cracking rate was 30%. Dynamic exclusion was implemented to remove some unnecessary information in MS/MS spectra.

### 3.5. GC-MS Analysis of VOCs

Tea volatile compound analysis was carried out using a Clarus SQ 8T gas chromatograph-mass spectrometer (Perkin Elmer, New York, NY, USA) equipped with an Elite-5MS column (30.0 m × 0.25 mm × 0.25 μm; Perkin Elmer). The GC-MS analytical procedure was conducted according to our previously published protocol [18]. The column temperature program was as follows: initial temperature 50 °C (hold for 5 min); increased to 125 °C at a rate of 3 °C/min, and held for 2 min; and increased to 180 °C at a rate of 5 °C/min, and maintained for 3 min; then increased to 230 °C at a rate of 15 °C/min, and held for 5 min. The flow rate of the carrier gas (high-purity helium, 99.999%) was 1 mL/min. The MS spectrometer was operated in electron impact mode with the electron energy 70 eV and a scan range of *m*/*z* 45–500. The ion source and mass spectrum transfer-line temperatures were 230 °C and 250 °C, respectively. The volatile metabolites were identified by matching the National Institute of Standards and Technology (NIST) mass spectral database (match rate > 80%) and retention index (RI, determined by n-alkane C7-C40). The chemical structure, name, and odor of the volatile compounds were determined according to PubChem (https://pubchem.ncbi.nlm.nih.gov, accessed on 10 August 2023) and The Good Scents Company Information System (http://www.thegoodscentscompany.com, accessed on 10 August 2023). The content of all identified volatiles was calculated using the peak area normalization method.

### 3.6. Statistical Analysis

All data were presented as mean ± standard deviation (SD) of three replicates. The mean and standard deviation of the data were calculated by Microsoft Excel 2010. Duncan’s multiple range test was used to analyze significant differences among different processing steps by using SPSS 20.0 statistical software. TBtools (Version 1.046) was used to compare the content dynamics of non-volatile metabolites and volatile compounds during different processing. The principal component analysis (PCA), hierarchical cluster analysis (HCA), and partial least squares discrimination analysis (PLS-DA) among different processing samples were performed using SIMCA 14.1 software. The volcano map analysis and graphs of non-volatile and volatile differential metabolites were performed using Origin 2022b software.

## 4. Conclusions

In this study, the dynamic changes and accumulation of metabolites during the ECS of Aijiao oolong tea were determined by using non-targeted metabolomics analysis. Out of the identified 306 non-volatile metabolites and 85 volatile metabolites, 159 non-volatile metabolites and 42 volatile metabolites were screened out as key differential metabolites. The accumulations of most metabolites exhibited dynamic changes, while some amino acids, nucleosides, and organic acids accumulated significantly after the turning-over treatment. The evolution characteristics of 27 key precursors or transformed VOCs during the ECS of Aijiao oolong tea were clarified, and it was found that the synthesis of aroma substances was mainly concentrated in lipids as precursors and glycosides as precursor pathways. The results revealed the dynamic changes in metabolites in the ECS during the processing of Aijiao oolong tea and provided valuable information for the formation mechanism of the unique flavor quality of Aijiao oolong tea. However, the mechanisms underlying flavor formation must be further investigated to apply these results during oolong tea production for quality assurance and the potential improvement of processing methods.

## Data Availability

Data are contained within the article or Supplementary Materials.

## Supplementary Materials

The following supporting information can be downloaded at: https://www.mdpi.com/article/10.3390/plants13091249/s1, Table S1. The information on 306 non-volatile metabolites, Table S2. The information on 159 differential non-volatile metabolites, Table S3. The relative content of 85 volatile components, Table S4. The information on 42 differential volatile metabolites, Table S5. The 27 key precursors or transformed volatile components during Aijiao oolong tea processing. Figure S1. Scatter plot of non-volatile metabolites present in Aijiao oolong tea of FTL vs. WTL, WTL vs. ZTL, and ZTL vs. DTL (A), (B), and (C); OPLS-DA cross-validation results of non-volatile metabolites present in Aijiao oolong tea of FTL vs. WTL, WTL vs. ZTL, and ZTL vs. DTL (D), (E), and (F), Figure S2. Scatter plot of VOCs present in Aijiao oolong tea of FTL vs. WTL, WTL vs. ZTL, and ZTL vs. DTL (A), (B), and (C); OPLS-DA cross-validation results of VOCs present in Aijiao oolong tea of FTL vs. WTL, WTL vs. ZTL, and ZTL vs. DTL (D), (E), and (F).
