# Supplementary material for: Metabolite Profiling Reveals the Dynamic Changes in Non-Volatiles and Volatiles during the Enzymatic-Catalyzed Processing of Aijiao Oolong Tea
Source: Plants (Basel). 2024 Apr 30;13(9):1249. doi: 10.3390/plants13091249 (PMC11085110; doi:10.3390/plants13091249)
Supplement: Supplementary file 1 [file plants-13-01249-s001.zip › Supplementary figures.pdf]

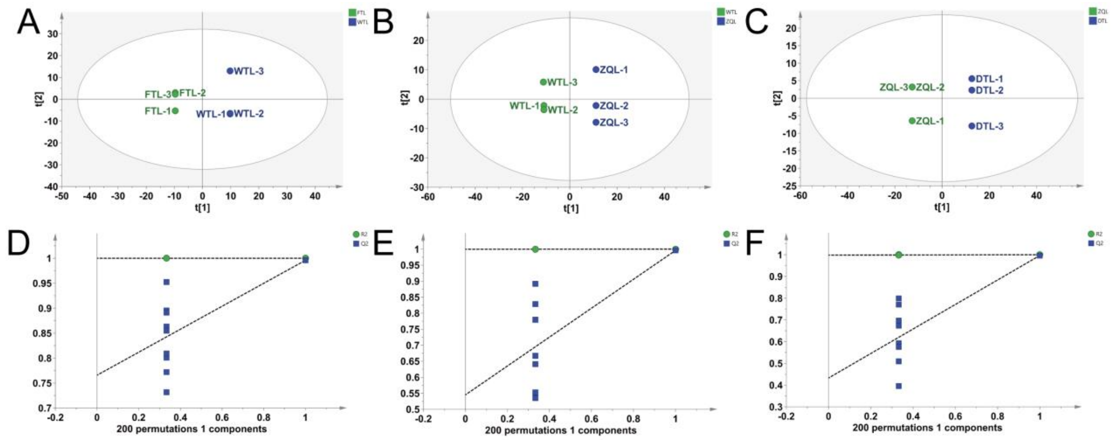

Figure S1. Scatter plot of non-volatile metabolites present in Aijiao oolong tea of FTL vs. WTL, WTL vs. ZTL, and ZTL vs. DTL (A), (B), and (C); OPLS-DA cross-validation results of on-volatile metabolites present in Aijiao oolong tea of FTL vs. WTL, WTL vs. ZTL, and ZTL vs. DTL (D), (E), and (F).

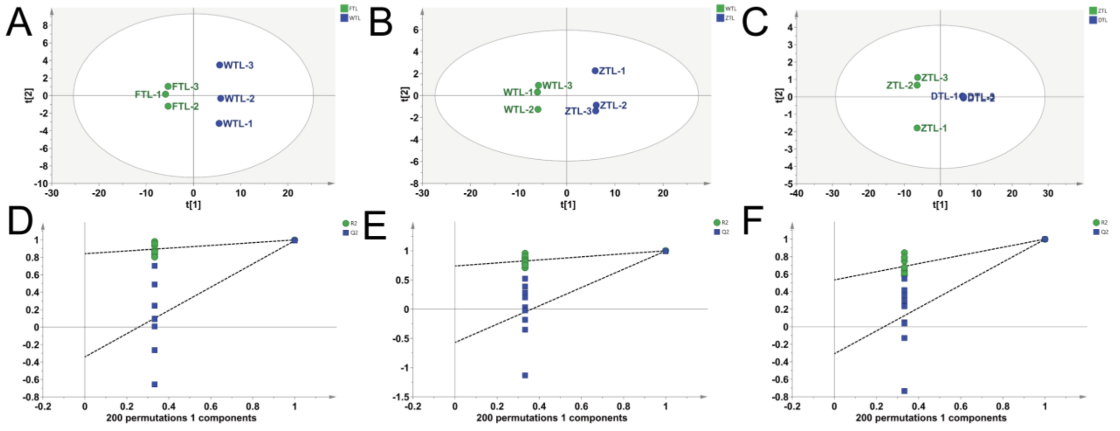

Figure S2. Scatter plot of VOCs present in Aijiao oolong tea of FTL vs. WTL, WTL vs. ZTL, and ZTL vs. DTL (A), (B), and (C); OPLS-DA cross-validation results of VOCs present in Aijiao oolong tea of FTL vs. WTL, WTL vs. ZTL, and ZTL vs. DTL (D), (E), and (F).
